# Experimental Parameters Influence the Observed Antimicrobial Response of Oak Wood (*Quercus petraea*)

**DOI:** 10.3390/antibiotics9090535

**Published:** 2020-08-24

**Authors:** Muhammad Tanveer Munir, Hélène Pailhories, Matthieu Eveillard, Mark Irle, Florence Aviat, Michel Federighi, Christophe Belloncle

**Affiliations:** 1 Laboratoire Innovation Matériau Bois Habitat Apprentissage (LIMBHA), Ecole Supérieure du Bois, 7 rue Christian Pauc, 44000 Nantes, France; tanveer.munir@esb-campus.fr (M.T.M.); mark.irle@esb-campus.fr (M.I.); 2 Laboratoire HIFIH, UPRES EA3859, SFR 4208, Université d’Angers, 49933 Angers, France; helene.pailhories@chu-angers.fr; 3 Laboratoire de Bactériologie, CHU Angers, 4 rue Larrey, 49933 Angers, France; MaEveillard@chu-angers.fr; 4 CRCINA and Inserm at Université de Nantes and Université d’Angers, 49933 Angers, France; 5 Your ResearcH-Bio-Scientific, 307 la Gauterie, 44430 Le Landreau, France; florenceaviat@gmail.com; 6 UMR INRA 1014 SECALIM, Oniris, Route de Gachet, CS 40706, CEDEX 03, 44307 Nantes, France; michel.federighi@oniris-nantes.fr

**Keywords:** agar diffusion, antimicrobial activity, wood, *Staphylococcus aureus*, *Acinetobacter baumannii*

## Abstract

The present investigation aimed to utilize a direct wood disc diffusion method to study the influence of plane of cutting, cutting method, sterilization method, and origin of tree on the antimicrobial activity of wood material. Six oak wood trees (*Quercus petraea*) were collected from 3 different locations in France. They were cut into 4 mm thick slices with either transverse (RT), tangential (LT) or radial (LR) faces. Round discs (diameter 9.95 ± 0.1 mm) were cut from the slices via a laser machine or a manual punch machine, and were sterilized with gamma irradiation (25 kGy) or autoclaving (121 °C). The antimicrobial activity of wood was tested using a direct diffusion method against *Staphylococcus aureus* and *Acinetobacter baumannii* isolates. The zone of inhibition around the wooden disc was recorded following the recommendations used for antibiotics tests. The results showed that *S. aureus* was more susceptible than *A. baumannii*, to the chemicals that diffused from the wood. The transverse face discs exhibited higher antimicrobial activity. Samples that had been sterilized by autoclaving showed significantly (*p* < 0.05) lower antimicrobial activity, whereas the cutting method and origin of tree did not influence the antimicrobial activity of wood material. Therefore, the choice of sterilization method and cutting planes must be taken into account while studying and interpreting the antibacterial properties of wood material.

## 1. Introduction

Certain wood species show antimicrobial activities against pathogenic microorganisms [1,2,3]. Therefore, they can be considered for applications in hygienically important places, such as in the food industry as food preparation, fermentation, and packaging material, in the livestock sector for animal bedding, and for the indoor construction of residential buildings including healthcare institutes [4,5,6,7,8]. The antimicrobial characteristics of wood material are attributed to its physical structure and chemical composition, which can create unsuitable survival conditions for different microbes [9]. As this physiochemical profile differs between wood species, the antimicrobial activity also varies accordingly [10,11]. For example, some wood extractives contain active antimicrobial compounds, such as tannins and phenolics; however, their presence and distribution vary in different wood species, and even in different parts of the same tree [1,12]. Similarly, the anatomy of the wood species and the way it is cut will alter the characteristics of the exposed surfaces, which in turn, can have an impact on the survival conditions for microbes [9].

Antimicrobial properties of wood are generally tested via extractive based methods, i.e., various solvents are used to remove compounds naturally found in wood [1,2,10,13]. The extracted compounds can be used to obtain a quantitative measure of antimicrobial action [14]. Unfortunately, extraction adds an extra step that involves chemical handling, which might alter the active ingredient [11]. Furthermore, these methods are limited to chemical profile testing, and the role of wood anatomy is not taken into account. A direct wood disc-based agar diffusion method (antiboisgram, a modified form of antibiogram which uses wooden discs instead of antibiotic discs) was recently developed that avoids these difficulties [3]. This method is based on the diffusion of chemicals from the wood disc into agar and observing the impact, if any, on the microbial growth [1,15].

A range of variables linked to wood could influence its physical and chemical composition, and subsequently, the antimicrobial potential of wood material [9]. The first factor is the anatomy of a tree which causes the wood within it to have three distinct faces depending on how it is cut: transverse (where the open ends of cells can be seen, known as transversal (radial-tangential, RT); longitudinal tangential (LT) and longitudinal radial (LR). Mixed faces also occur in the real world. These faces will have different surface roughness and diffusion coefficients which are expected to change the antimicrobial behavior of material. For example, Pailhories et al. [15] reported that oak wood (*Quercus* sp.) showed different antimicrobial activities against various isolates of *Staphylococcus aureus* depending upon the planes of cutting. The second factor is the origin of the tree because the composition of wood extractives may vary in different trees of the same species depending upon where they grew [12,16]; thus, their antimicrobial activity could also change accordingly. The third factor is the condition of wood which, in terms of aging, erosion, tearing, and weathering, can influence the physical structure and chemical composition and, likewise, the antimicrobial potential of this material. The condition of wood can be affected by cleaning and sterilization methods [17], such as when food preparation boards are sterilized using hot water washing and ultraviolet rays [9,11], even gamma radiations at a dose of 25–50 kGy are used to sterilize the wooden samples in experimental studies [3,18]. The final factor to consider is the cutting of wood using high energy laser beams, which burn the material on contact areas. Yet, there is no evidence available on the influence of such treatments on the antimicrobial behavior of wood. Studying the influence of all these variables on the antimicrobial activity of wood is necessary to better employ this material in hygienically sensitive places.

This study aimed to test the dependence of antimicrobial activity on wood origin, sterilization method, cutting method, and the plane of cutting. To our knowledge, the effect of the aforementioned variables has not been documented before.

## 2. Materials and Methods

### 2.1. Wood

Oak wood (*Quercus petraea*) was selected for this study because of its known durability and antimicrobial behavior. This wood was obtained from six trees growing in Département de la Sarthe in the Région Pays de la Loire of France and having three different soil types. Logs were cut 1.30 m above ground. The wood went through a natural drying process of 6 weeks until moisture content reached 12%.

### 2.2. Wood Discs Preparation

The logs were initially cut into boards of 40 mm thickness and freed from sapwood and pith. Then boards were further cut by the electric saw (Altendorf-F45, Minden, Germany) into thinner (3.5–4 mm) sheets with respect to transversal (RT), tangential (LT), and radial (LR) planes. These veneers were used to prepare uniform-sized circular discs (diameter 9.95 ± 0.1 mm and weighing 213 ± 15 mg) using a laser cutting machine (Trotec-SP500 C60, Wels, Austria) or a manual punch machine (SyretteSyderic ET23N). The samples were conditioned to ~12% moisture content in a climatic chamber.

### 2.3. Sterilization of Samples

The discs were packed in sterile plastic bags and divided into three groups. One group was kept unsterilized (N), and other two were sterilized by either gamma irradiation (G) at 25 kGy (Ionisos, Sablé sur Sarthe, France) or by autoclaving (A) at 121 °C and 100 kPa for 15 min (VAPOUR-Line Lite, VWR).

### 2.4. Bacteria

One reference strain of a Gram-positive bacteria, *Staphylococcus aureus* ATCC 29213, and a clinical isolate of a Gram-negative bacteria *Acinetobacter baumannii* carrying OXA-23 carbapenemase gene were obtained from the University Hospital of Angers, France. Both these bacteria were selected because of their importance in causing nosocomial infections in the healthcare facilities, plus *S. aureus* can also cause foodborne infections when cross-contaminated from food contact surfaces.

### 2.5. Agar Diffusion Method for Antiboisgram (Modified Antibiogram)

**Figure 1:**
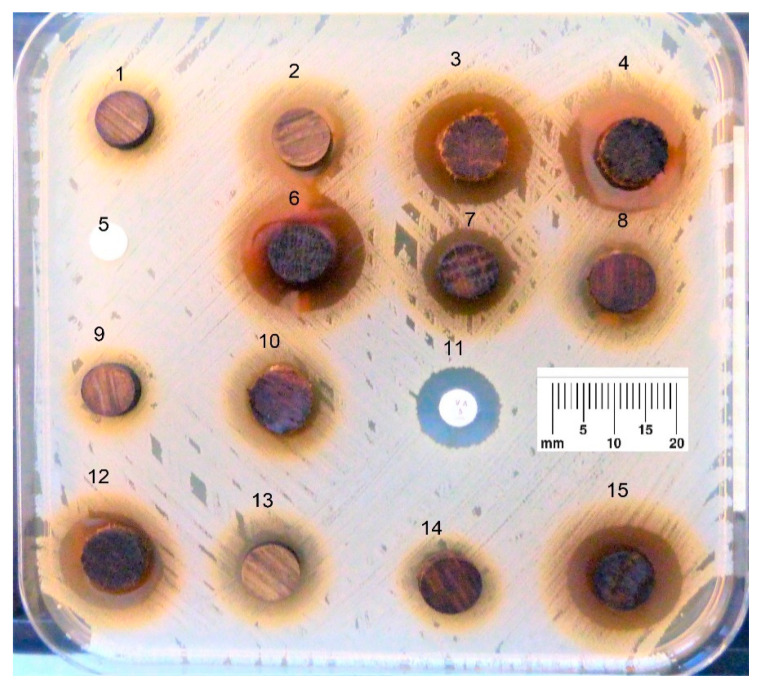
Antibiogram of *Staphylococcus aureus* streaked on Mueller–Hinton agar plate with oak wood (diameter 9.95 ± 0.1 mm) and control paper discs (diameter 6 mm). (1) Tree 1, laser cut, radial face, autoclaved; (2) Tree 3, laser cut, radial face, gamma irradiated; (3) Tree 2, manual cut, transversal face, gamma irradiated; (4) Tree 1, manual cut, transversal face, non-sterilized; (5) Inert filter paper disc—negative control; (6) Tree 1, laser cut, transversal face, gamma irradiated; (7) Tree 2, laser cut, transversal face, autoclaved; (8) Tree 3, manual cut, radial face, gamma irradiated; (9) Tree 3, laser cut, radial face, autoclaved; (10) Tree 2, manual cut, radial face, gamma irradiated; (11) Vancomycin 5 μg—positive control; (12) Tree 2, manual cut, transversal face, non-sterilized; (13) Tree 1, laser cut, radial face, gamma irradiated; (14) Tree 3, manual cut, radial face, autoclaved; (15) Tree 2, laser cut, transversal face, gamma irradiated; the scale is in millimeters.

Direct disc diffusion method was used as described in earlier studies [3,15]; briefly, the bacterial suspensions adjusted to a density of 0.5 McFarland were inoculated by streaking on Mueller–Hinton agar plates (12 × 12 cm) (BioRad, Marnes La Coquette, France), according to the recommendations of the Antibiogram Committee of the French Society of Microbiology [19]. The wood discs were then placed directly on the agar. A total of 16 samples could be tested on each plate (Figure 1). The plates were incubated at 37 °C for 24 h. Positive control discs (diameter 6 mm) were purchased (Oxoid, Basingstoke, UK) and included Vancomycine (5 µg) for *S. aureus* and Colistine (25 µg) for *A. baumannii*. The negative control, inert filter paper discs (diameter 6 mm), were also prepared commercially (Bio-Rad, Marne-la-Coquette France).

The inhibition zones, as defined by CASFM (Comité de l’antibiogramme de la Société Française de Microbiologie) and EUCAST (European Committee on Antimicrobial Susceptibility Testing) [19], were manually measured after 24 h of incubation (Figure 1). The tests were carried out in triplicate and all the experiments were conducted in the laboratory of bacteriology of the University Hospital of Angers, France.

### 2.6. Statistical Analysis

Data were analyzed by one-way analysis of variance (ANOVA), followed by measurement of statistically significant differences using Dunnett’s post hoc test (SAS Institute Inc., Cary, NC, USA). All data are presented as the mean ± standard deviation (SD). The results with *p* < 0.05 were considered statistically significant.

## 3. Results

**Figure 2:**
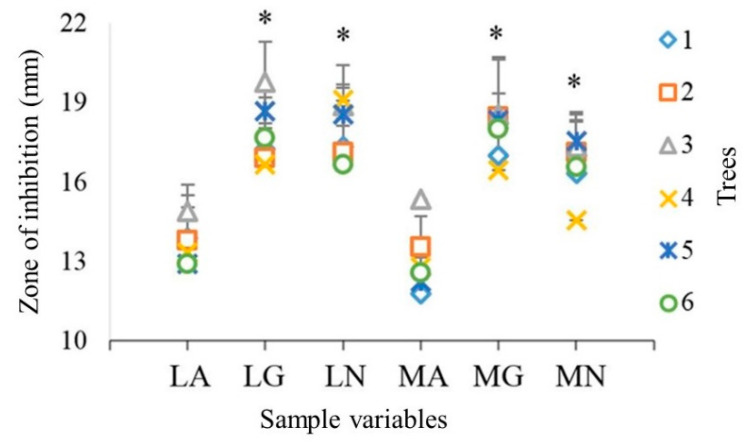
Effect sterilization on the antimicrobial activity of different trees against *Staphylococcus aureus*. Y axis is the mean ± standard deviation (*n* = 3) of zone of inhibition (mm) including the diameter of wooden discs (9.95 ± 0.1 mm); LA = Laser cut autoclaved samples; LG = Laser cut gamma irradiated samples; LN = Laser cut non-sterilized samples; MA = Manual cut autoclaved samples; MG = Manual cut gamma irradiated samples; MN = Manual cut non-sterilized samples; 1–6 number of trees; * *p* < 0.05.

**Figure 3:**
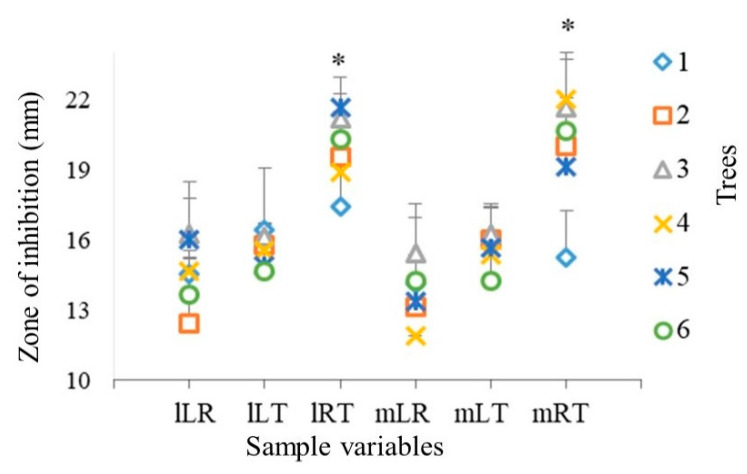
Effect cutting planes on the antimicrobial activity of 6 trees against *Staphylococcus aureus*. *Y*-axis is the value of zone of inhibition including the diameter of wooden discs (9.95 ± 0.1 mm); lLR = Laser cut radial plane samples; lLT = Laser cut tangential plane samples; lRT = Laser cut transversal plane samples; mLR = Manual cut radial plane samples; mLT = Manual cut tangential plane samples; mRT = Manual cut transversal plane samples; 1–6 number of trees; * *p* < 0.05.

Antibiosgram results showed that the variation of activity among six trees and three different locations was non-significant (*p* < 0.05) in all the experiments (Figure 2 and Figure 3). Tree 1 showed an exception in case of transversal face (lRT and mRT in Figure 3) which had slightly lower activity as compared to the other five trees.

The results of the effect of different sterilization techniques on the antimicrobial activity of six oak trees are shown in Figure 2. Autoclaved samples (LA, MA), irrespective of preparation method, had significantly (*p* < 0.05) lower antimicrobial activity against *S. aureus* as compared to non-sterilized (LN and MN) and gamma sterilized samples (LM and MG). There was no significant difference (*p* < 0.05) between the activities of non-sterilized and gamma sterilized samples.

Similar trends were observed in the case of *A. baumannii*, with higher variation and resistance of bacteria. The gamma-irradiated and non-sterilized samples showed higher antimicrobial activity as compared to autoclaved samples. However, results were significant at *p* < 0.05 (Figures S1 and S2).

Figure 3 shows that planes of cutting affected the antimicrobial activity of wooden discs, with the highest activity being for RT cutting, followed by LT and LR. The statistical analysis confirmed that the effect of cutting planes on the antimicrobial activity of oak wood against *S. aureus* in the RT cuttings was significantly (*p* < 0.05) higher as compared to LT and LR, however, there was no significant difference of activity between two longitudinally cut planes *viz* LT and LR (Figure 3).

The two-disc preparation/cutting methods, manual, and laser, had a non-significant (*p* < 0.05) effect on the antimicrobial activity of tested wood (Figure 2 and Figure 3).

## 4. Discussion

The results of this study regarding the antimicrobial activity of wood material are supported by the previous publications reporting that different woods, including oak [15], larch [1], pine [2], spruce [13], beech, and fir [3], have antimicrobial properties against various isolates of *S. aureus*. In this study, however, the antimicrobial activity of wood material was very low against *A. baumannii*, as compared to *S. aureus*. This difference could be attributed to the difference in cell wall types of *S. aureus* (Gram-positive) and *A. baumannii* (Gram-negative) bacteria. This reason is more likely because the mechanism of action of most of the wood antimicrobial compounds, including flavonoids, tannins, aldehydes, phenolic acids, terpenoids, alkaloids, and terpenes, is to damage the cell wall of bacteria [9].

No significant difference in the antimicrobial activity among the six trees (Figure 2 and Figure 3) was observed, even though they originated from three different locations (Table 1). On the basis of these findings, we can hypothesize that all the trees had similar levels of anti-microbial compounds. Earlier studies have also reported that ecological zone has a negligible effect on the chemical composition of different oak species, e.g., *Q. robur* and *Q. petraea* [16]. This uniformity may also be due to the fact that similar parts of the wood were collected from different trees (mature heartwood), which reduced the chances of high variations in chemical composition. Additionally, it is possible that the total extractive content varied among different trees, however, the active antimicrobial ingredients were similar in all the trees. Furthermore, possibility of a lack of variations among groups might be owing to the low sensitivity of this method, which could not identify these minor variations.

The wood discs with transverse (RT plane) faces showed stronger antimicrobial activity as compared to both longitudinal faces (LT and LR). These findings are in line with the earlier reports [3,15,20]. In those publications, this variation of activity was attributed to the arrangement of fibers in wood. The fibers in wood mostly run longitudinally and the diffusion in the longitudinal direction is about 10 to 15 times faster than tangential or radial diffusion [21]; therefore, when the RT faced discs were placed on agar, there is potentially a higher diffusion of active compounds into agar than from the LT and LR samples. Consequently, the wood cut into RT direction would be more efficient to control the microbial growth. The difference among the activity of LT and LR cutting was non-significant which could be due to minor differences of fiber arrangement in both cuttings and the current method did not sensitively measure this difference.

The antimicrobial activities of non-sterilized and gamma sterilized samples were similar as previously described [3]. It shows that even if the non-sterilized samples are contaminated with other microbes [18], this method is not influenced by their presence, especially, when used for screening [3]. Otherwise, in a natural environment, many microbial interactions occur on wooden surfaces, which could be antagonistic or symbiotic, and can interplay with the antimicrobial activity of wood against specific microbes. For example, the studies have shown that the wood being an acidic material supports the growth of lactic acid bacteria which can counter the growth of some bacterial pathogens, e.g., *Listeria, Salmonella* and *Campylobacter* spp. [22,23,24]. In addition, researchers have used certain wood microbes, e.g., *Bacillus subtilis,* to treat surfaces and stop the growth of undesired wood degrading fungi [25,26,27].

The lower antimicrobial activity of autoclaved wood samples, as compared to non-sterilized and gamma sterilized wood, shows the loss of some active compounds. As many of the volatile organic compounds (VOCs) are responsible for the antimicrobial action of wood [13], their loss by high temperature treatment [10,28] might have reduced this ability. This finding is important because the loss of VOCs from wood continues throughout the usage life until reaching a minimum level. In such conditions, the antimicrobial activity is also likely to reduce over time. In an earlier study, it was observed that the two year storage of wooden discs in polythene bags did not reduce the antimicrobial activity of samples [3]; thus, we can assume that the lost molecules degraded faster at the higher temperature (of autoclaving) than at room conditions. It is not clear, however, if the autoclaving changed the physical structures of wood, leading to reduced diffusion and ultimately, the antimicrobial activity.

Contrary to loss of antimicrobial activity by the heat of autoclaving, the laser cutting and manual cutting did not differ in antimicrobial action. The laser cuts the wood samples only on the sides, and not the contact surface, which is supposed to be tested for antimicrobial action. Therefore, it can be assumed that there are no new chemicals formed or reduced to interfere with the antimicrobial activity. In earlier studies, laser cutting has been described as an efficient and quicker method for uniformly sized sample preparation, as compared to manual sawing [3,15]. Further research should be targeted to study the influence of surface burning techniques on antimicrobial activity also because this technique is becoming very common in the furniture industry.

The method used in this study has a limitation of variability when the antimicrobial activity is low. For example, the minimum detection limit of the direct diffusion method was considered as 1 mm (10 + 1 mm) of the zone of inhibition and in the case of *A. baumannii,* the activity was very low as compared to *S. aureus*. Consequently, the influence of wood variables in the case of *A. baumannii* was non-significant when considering *p* < 0.05. Therefore, future studies should consider this point when interpreting the results.

## 5. Conclusions

The findings show that the direct diffusion method can be used to elaborate the antimicrobial properties of wood material. In addition, some influencing variables, such as the effect of sterilization and cutting planes, should be taken into consideration when testing the antimicrobial properties of wood material. Based on the sampling set, antibacterial properties of oak (*Quercus petraea)* tree were found to be neither origin nor tree dependent. Future studies should apply this methodology with uniform-sized and weighed discs to confirm these variables in multiples wood species and different microbes.

## Figures and Tables

**Table 1:** Characteristics of oak trees (*Quercus petreae*) from Région Pays de la Loire, France.

Specimen Number	1 and 2	3 and 6	4 and 5
Area	Parcelle 10 A 4ha 48	Parcelle 402 F 6ha 48	Parcelle 21E 4ha 37
Coordinates	47°51′18.4′′ N 0°08′25.5′′ W	47°45′35.2′′ N 0°16′51.3′′ W	47°50′38.4′′ N 0°11′28.0′′ W
Age (years)	90	80	100
Soil fertility	Medium	Good	Medium
Total Height (m)	30	30	33

## Supplementary Materials

The following are available online at https://www.mdpi.com/2079-6382/9/9/535/s1, Figure S1: Effect sterilization on the antimicrobial activity of different trees against *Acinetobacter baumannii*, and Figure S2: Effect cutting planes on the antimicrobial activity of 6 trees against *Acinetobacter baumannii*.
