# Supplementary material for: Experimental Parameters Influence the Observed Antimicrobial Response of Oak Wood (Quercus petraea)
Source: Antibiotics (Basel). 2020 Aug 24;9(9):535. doi: 10.3390/antibiotics9090535 (PMC7558063; doi:10.3390/antibiotics9090535)
Supplement: Supplementary file 1 [file antibiotics-09-00535-s001.pdf]

antibiotics

MDPI

Supplementary material

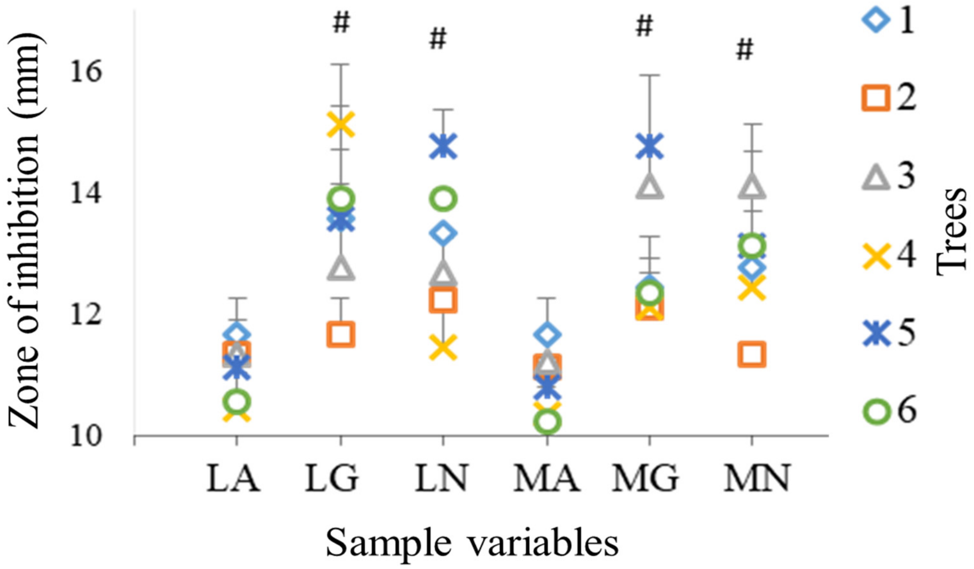

Figure S1. Effect sterilization on the antimicrobial activity of different trees against *Acinetobacter baumannii*. Y axis is the mean ± standard deviation (n=3) of zone of inhibition (mm) including the diameter of wooden discs (10 mm); LA = Laser cut autoclaved; LG = Laser cut gamma irradiated; LN = Laser cut non-sterilized; MA = Manual cut autoclaved; MG = Manual cut gamma irradiated; MN = Manual cut non-sterilized; 1 - 6 number of trees; # $p < 0.1$.

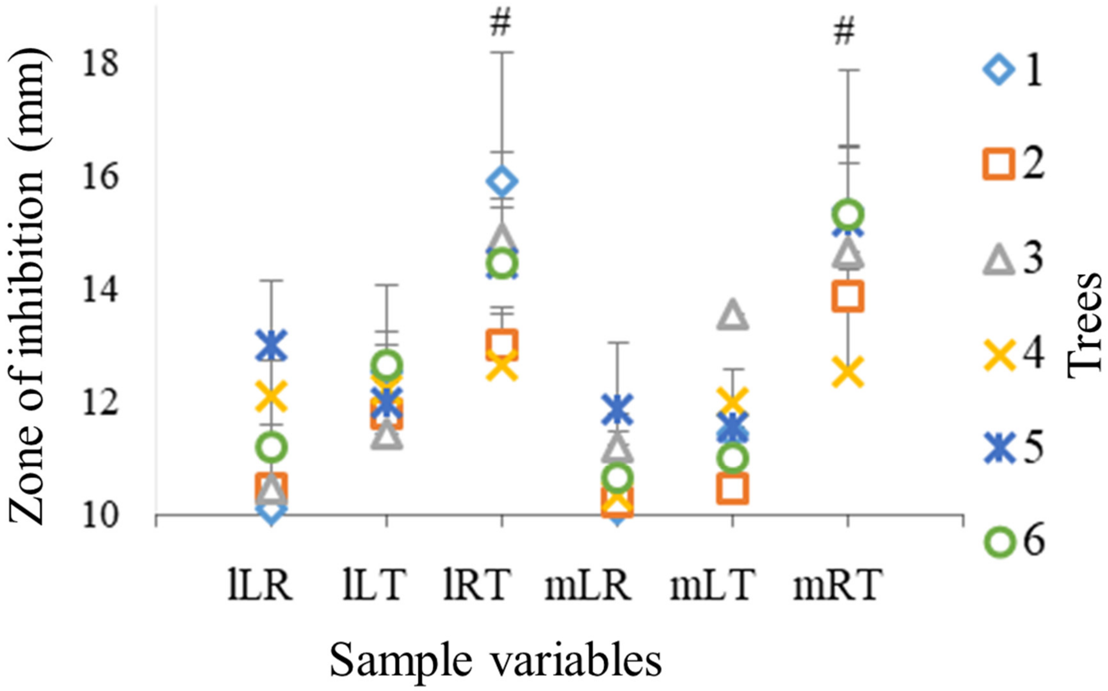

Figure S2. Effect cutting planes on the antimicrobial activity of 6 trees against *Acinetobacter baumannii*. Y axis is the value of zone of inhibition including the diameter of wooden discs (9.95±0.1 mm). lLR = Laser cut radial plane samples; lLT = Laser cut tangential plane samples; lRT = Laser cut transversal plane samples; mLR = Manual cut radial plane samples; mLT = Manual cut tangential plane samples; mRT = Manual cut transversal plane samples; 1 - 6 number of trees; # $p < 0.1$.
